# Exploring the Human Plasma Proteome for Humoral Mediators of Remote Ischemic Preconditioning - A Word of Caution

**DOI:** 10.1371/journal.pone.0109279

**Published:** 2014-10-15

**Authors:** Erik Helgeland, Lars Ertesvåg Breivik, Marc Vaudel, Øyvind Sverre Svendsen, Hilde Garberg, Jan Erik Nordrehaug, Frode Steingrimsen Berven, Anne Kristine Jonassen

**Affiliations:** 1 Department of Biomedicine, Faculty of Medicine and Dentistry, University of Bergen, Bergen, Norway; 2 Department of Clinical Science, Faculty of Medicine and Dentistry, University of Bergen, Bergen, Norway; 3 Department of Anaesthesia and Surgical Services, Haukeland University Hospital, Bergen, Norway; University of Louisville, United States of America

## Abstract

Despite major advances in early revascularization techniques, cardiovascular diseases are still the leading cause of death worldwide, and myocardial infarctions contribute heavily to this. Over the past decades, it has become apparent that reperfusion of blood to a previously ischemic area of the heart causes damage in and of itself, and that this ischemia reperfusion induced injury can be reduced by up to 50% by mechanical manipulation of the blood flow to the heart. The recent discovery of remote ischemic preconditioning (RIPC) provides a non-invasive approach of inducing this cardioprotection at a distance. Finding its endogenous mediators and their operative mode is an important step toward increasing the ischemic tolerance. The release of humoral factor(s) upon RIPC was recently demonstrated and several candidate proteins were published as possible mediators of the cardioprotection. Before clinical applicability, these potential biomarkers and their efficiency must be validated, a task made challenging by the large heterogeneity in reported data and results. Here, in an attempt to reproduce and provide more experimental data on these mediators, we conducted an unbiased in-depth analysis of the human plasma proteome before and after RIPC. From the 68 protein markers reported in the literature, only 28 could be mapped to manually reviewed (Swiss-Prot) protein sequences. 23 of them were monitored in our untargeted experiment. However, their significant regulation could not be reproducibly estimated. In fact, among the 394 plasma proteins we accurately quantified, no significant regulation could be confidently and reproducibly assessed. This indicates that it is difficult to both monitor and reproduce published data from experiments exploring for RIPC induced plasma proteomic regulations, and suggests that further work should be directed towards small humoral factors. To simplify this task, we made our proteomic dataset available via ProteomeXchange, where scientists can mine for novel potential targets.

## Introduction

Remote ischemic preconditioning (RIPC) is an emerging treatment for reducing ischemia reperfusion injury (IRI) in the heart. Several proof-of-concept studies and small randomized controlled trials have demonstrated that the human heart is amenable to RIPC [1]–[5]. Recently, it was also reported that 3 cycles of 5 min upper arm ischemia substantially reduced myocardial injury after coronary artery bypass graft in large pools of patients. This was demonstrated by a significant decrease in cardiac troponin I (cTnI) release, significantly improving prognosis with a reduction in mortality in the group receiving RIPC compared to controls [6].

The signaling pathway of RIPC in the human heart is just starting to be uncovered [7], but how the RIPC stimulus is transferred from the arm to the heart remains unclear. Compelling preclinical evidence suggests communication via one or more unknown humoral factors: First, it seems that a period of reperfusion after the RIPC stimulus is required for protection, suggesting that wash-out of blood borne factors and transport to the site of protection is involved [8]–[10]. Secondly, it was demonstrated that effluent from preconditioned hearts could transfer the protection to naïve recipient hearts and that the protection is mediated via small, unknown hydrophobic factors of protein nature between 3.5 and 15–30 kDa [11]–[16]. Moreover, the fact that this humoral factor is effective at a remote location after dilution in blood or perfusion fluid, hints at a large concentration change which should be detectable by modern proteomic techniques.

In fact, several proteins were recently found to be regulated after RIPC, paving the way for potential use of these cardioprotective compounds in the clinic. Notably, Hepponstall *et al.* conducted an ambitious study where 806 differentially expressed peptides were identified after RIPC. Among them, 133 could be mapped to 48 protein sequences [17]. In addition, 2D gel experiments reported 33 regulated spots with 6 identifiable proteins. Surprisingly, only one of the given protein accession numbers could be found in both the mass spectrometry and the 2D gel result sets. Subsequently, Pang *et al.* found 14 proteins to be differentially regulated by RIPC and validated these findings by Western blotting [18]. Notably, only one of the accessions reported, Gelsolin (UniProt accession number P06396), could be mapped to those published by Hepponstall *et al.* Additionally, albeit with different sequences, both studies underlined the regulation of Apolipoprotein A-I. However, Pang *et al.* found this protein to be down-regulated, while Hepponstall *et al.* found it down-regulated using gels and up-regulated when applying a gel free technique in the same study. The latter result is in concordance with the study of Hibert *et al.* showing a 30% up-regulation of Apolipoprotein A-I, postulating it to be the principal factor behind RIPC mediated cardioprotection [19]. Later on, however, Hilbert *et al.* published another report where this protein appeared not to be regulated, but seven other related proteins presented a Mann-Whitney test p-value <0.05 [20]. Notably, no protein passed a more stringent 0.01 threshold and all proteins showed moderate regulations (between 0.58∶1 and 1.2∶1), consistently below the two fold change regulation level generally used in biology. The results of Davidson *et al.* suggested SDF-1α to be the main mediator of RIPC, presumably communicating the cardioprotection via SDF-1α/CXCR4 signaling [21]. Importantly, Przyklenk [22] criticized the latter study for its limitations, as the plasma levels of SDF-1α should have been monitored both before and after the RIPC stimulus, thereby failing to validate the factor as a mediator of RIPC.

Discovery studies “often significantly overestimate their findings” as claimed by Gosho *et al.* [23]. Consequently, prior to clinical testing, protein markers must be validated, preferably using targeted mass spectrometry based proteomics [24]. For that, quantitative assays are built in order to target and quantify the compounds of interest. Setting up such an assay requires experimental data on the digestion, peptide elution, ionization, and fragmentation profile of the targeted protein. This information is, however, not available from any of the mentioned mass spectrometry datasets. Notably, they are not available in public repositories, despite this being standard publication guidelines in proteomics [25],[26]. In fact, the peptide information is not available and in the case of Hepponstall *et al.*, not even the estimated protein ratios are reported.

The disagreement in the literature, even in studies from the same group or within the same study, together with the lack of provided information, show the striking need for a transparent proteomic dataset stringently monitoring the proteomic changes induced by RIPC. Here, we present a quantitative in depth sequencing of the plasma proteome before and after RIPC. Six healthy donors underwent RIPC according to the protocol used in consensus with the literature and whose efficiency has been long established. In contrast to previously reported studies, the digested plasma samples were multiplexed using isobaric tags and fractionated, thereby limiting inter-sample artifacts and dramatically increasing sample coverage. The protein identification and quantification was achieved using open source software applying stringent quality criteria to avoid reporting artifact regulations. The acquired raw data was deposited to the ProteomeXchange consortium [27] together with identification results, and can thereby freely be accessed, inspected and even reprocessed to better plan further experiments [28].

## Materials and Methods

**Figure 1:**
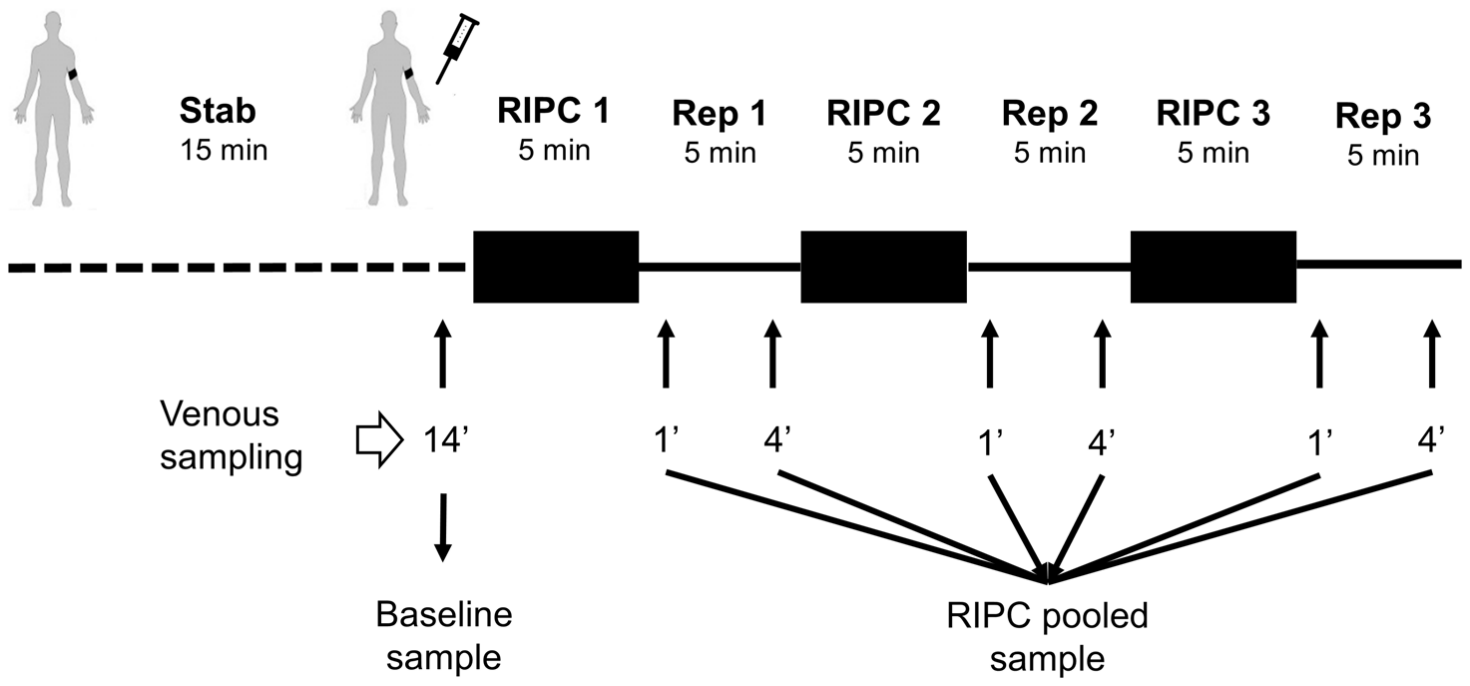
Experimental protocol. A peripheral venous catheter was inserted in the cubital fossa of subject for blood sampling. The subject rested for 14 min reclined on a bench before the baseline sample was drawn. The blood pressure cuff was inflated to 200 mmHg for 5 min before being released. Blood samples were drawn at 1 and 4 min into reperfusion from the ipsilateral arm. Blood samples were centrifuged to collect plasma which was stored at −80°C. Before analysis, all six reperfusion samples were pooled for each subject.

This study was approved by the regional ethics committee for medical and health research in Western Norway (REK 2010/1642-1). Written informed consent was obtained from all participants and the study conformed to the principles in the declaration of Helsinki. Six healthy adult male donors aged 29±2 years old (mean ± SD), not on any medication, underwent the RIPC protocol which consisted of 3 cycles of 5 min upper arm ischemia alternating with 5 min reperfusion (Figure 1). All subjects rested upright on a bench for 15 min before ischemia was induced by inflating a blood pressure cuff to 200 mmHg. Venous blood samples were collected from the ipsilateral arm after 14 min of rest in the beginning, and at 1 and 4 min into each reperfusion period. Blood samples were collected in K_2_ EDTA-coated tubes (Vacutainer, BD). Samples were centrifuged at 1,500 rcf at 4°C for 15 min within 30 min after sampling. 1.5 ml plasma was transferred to Eppendorf tubes before a second centrifugation at 15,000 rcf at 4°C for 15 min to remove any cell contaminants or cell fragments. ∼90% of the clear supernatant was removed using a 1 ml pipette and stored at −80°C. The whole process from sampling to freezing of each individual sample took less than 75 min. Samples from 1 and 4 min of each of the reperfusion periods were pooled, resulting in 12 samples: a sample before (control) and after RIPC for each of the six individuals.

### Chemicals

Trypsin was purchased from Promega. N-octyl-β-D-glycopyranoside (NOG), acetonitrile (ACN), formic acid (FA), ammonium formate and water were purchased from Sigma-Aldrich. Water and ACN were of HPLC quality.

### Abundant protein depletion and concentration

20 µl of plasma from each sample was depleted using a human Multiple Affinity Removal System (MARS Hu-14) 4.6 mm×50 mm LC column (Agilent Technologies) according to the protocol provided by the supplier, using a Dionex 3000-series LC system. The MARS column depletes the plasma of albumin, IgG, antitrypsin, IgA, transferrin, haptoglobin, fibrinogen, alpha-2-macroglobulin, alpha-1-acid glycoprotein, IgM, apolipoprotein AI, apolipoprotein AII, complement C3 and transthyretin. The protein depleted plasma samples were concentrated using 3 kDa ultracentrifugation filters (Amicon Ultra-4, Millipore, Bedford, MA) pre-treated with 0.1% NOG.

### Protein digestion and iTRAQ labeling

The entire depleted protein sample was reduced, cysteine blocked, trypsin digested (1∶20, trypsin∶protein, w/w), iTRAQ labeled (114, 115, 116 and 117) and combined according to the protocol using the chemicals provided (AB Sciex). The iTRAQ 4-Plex kit allowed us to multiplex two conditions from two donors per kit, resulting in three parallel experiments as detailed in Table 1. Both conditions (before and after RIPC) followed the exact same downstream workflow for every individual.

**Table 1:** Repartition on every iTRAQ channel of the samples at baseline and after RIPC for the six donors.

iTRAQ Channel	Experiment 1	Experiment 2	Experiment 3
**114**	Donor 1 baseline	Donor 3 RIPC	Donor 5 baseline
**115**	Donor 1 RIPC	Donor 3 baseline	Donor 5 RIPC
**116**	Donor 2 baseline	Donor 4 RIPC	Donor 6 baseline
**117**	Donor 2 RIPC	Donor 4 baseline	Donor 6 RIPC

### Mix-mode fractionation

iTRAQ labeled peptides were fractionated into 60 fractions using a mixed-mode (MM) reversed phase anion exchange (RP-AX) Sielc Promix column as described by Philips *et al.* [29] (MP-10.250.0530, 1.0×250 mm, 5 µm, 300 Å, Sielc Technologies, Prospect Heights, Illinois) coupled to an Agilent 1260 series LC system (Agilent Technologies, Palo Alto, CA). The iTRAQ labeled peptides were reconstituted in 20 mM ammonium formate, 3% ACN (buffer A) and loaded on the column in 85% buffer A for 10 minutes at a flowrate of 50 µl/min. The peptides were eluted from the column increasing the contents of buffer B (2 mM ammonium formate, 80% ACN, pH 3.0), from 15% to 60% in 35 minutes and further to 100% buffer over 10 minutes. Buffer B was held constant for 5 minutes before the column was equilibrated for 10 minutes in 85% buffer A. The fractions from the first 10 minutes of the gradient were discarded.

### LC-MS/MS analyses

Fifty fractions from the MM RP-AX separation from each sample were analyzed on an LTQ-Orbitrap Velos Pro (Thermo Scientific) coupled to a Dionex Ultimate NCR-3000RS LC system. The fractions were dissolved in 1% FA and trapped on the pre-column (Dionex, Acclaim PepMap 100, 2 cm×75 µm i.d, 3 µm C18 beads) in buffer A (2% ACN, 0.1% FA) at a flowrate of 5 µl/min for 5 minutes before separation by reverse phase chromatography (Dionex, Acclaim PepMap 100, 15 cm×75 µm i.d., 3 µm C18 beads) at a flow of 280 nL/min. The fractions were run on three nano LC gradients: The first fifteen fractions were run on a LC gradient consisting of a gradient starting at 5% buffer B (90%ACN, 0.1% FA) ramping to 12% buffer B over 55 minutes (5–60 min), the gradient was subsequently ramped to 30% buffer B in 30 minutes (60–90 min), increased to 90% B in 10 minutes (90–100 min), held for 5 minutes (100–105 min) followed by ramping to 5% buffer B for 3 minutes (105–108) and equilibration of the column in 12 minutes (108–120); fractions 16–35 were separated on the following LC gradient: 0–5.0 minutes 5% buffer B, 5.0–5.5 minutes 8% buffer B, 5.5–60 minutes 20% buffer, 60–90 minutes 35% buffer B; the last fractions (36–50) were separated using the following gradient: 0–5.0 minutes 5% buffer B, 5.0–5.5 minutes 8% buffer B, 5.5–90 minutes 40% buffer. The last part of the nano LC gradient is similar for all three gradients.

The mass spectrometer was operated in data-dependent-acquisition (DDA) mode to automatically switch between full scan MS and MS/MS acquisition. The instrument was controlled by Tune 2.6.0 and Xcalibur 2.1. Survey full scan MS spectra (from m/z 300 to 2,000) were acquired in the Orbitrap with resolution R = 60,000 at m/z 400 (after accumulation to a target value of 1E6 in the linear ion trap with maximum allowed ion accumulation time of 500 ms). The 7 most intense eluting peptides above an ion threshold of 1,000 counts and charge states 2 or higher, were sequentially isolated in the high-pressure linear ion trap to a target value of 5E5 at a maximum allowed accumulation time of 1,000 ms, and isolation width maintained at 2 Da. Fragmentation in the Higher-Energy Collision Dissociation (HCD) cell was performed with a normalized collision energy of 40%, and activation time of 0.1 ms. Fragments were detected in the Orbitrap at a resolution of 7,500 with first mass fixed at m/z 100.

### Data analysis

All RAW data were transformed into mgf peak lists using the ProteoWizard software [30] package version 2.2.2954. The obtained peak lists were searched with OMSSA [31] version 2.1.9 and X!Tandem [32] Cyclone 2013.2.01.1 using SearchGUI [33] version 1.12.2. Peak lists were searched against a concatenated target/decoy [34] version of the human complement of the UniProtKB/Swiss-Prot database [35] (downloaded on September 2012). The decoy sequences were created by reversing the target sequences in SearchGUI. Search settings were as follows: Trypsin with a maximum of 2 missed cleavages; 10 ppm as MS, 0.6 Da as MS/MS tolerances, respectively; fixed modifications: methylthio of Cys (+45.987721 Da) and iTRAQ on Lys and peptide N-term (+144.105918 Da); and variable modifications: oxidation of Met (+15.994915 Da) and iTRAQ on Tyr (+144.105918 Da). All other OMSSA or X!Tandem settings were kept at the default values set in SearchGUI. Peptides and proteins were inferred from the search engine results using PeptideShaker (http://peptide-shaker.googlecode.com) [36]. Peptide to Spectrum Matches (PSMs), peptides and proteins were validated at a stringent 1% FDR estimated using the decoy hits. The mass spectrometry proteomics data have been deposited to the ProteomeXchange Consortium(http://proteomecentral.proteomexchange.org) [27] via the PRIDE partner repository [37],[38] with the dataset identifier PXD000605 and DOI 10.6019/PXD000605.

For every validated protein, the iTRAQ reporter ions were extracted from spectra of validated PSMs and deisotoped using the isotope abundance matrix [39]. Intensities were normalized using the median intensity in order to limit the ratio deviation [40] and peptide and protein ratios were estimated using maximum likelihood estimators [41]. Ratios were log2 converted and normalized to the median to avoid inter-sample bias. Only those proteins presenting two or more validated and quantified peptides were retained for the quantitative analysis. Standard contaminants as well as all proteins with known affinity to the antibodies in the MARS column were excluded from downstream statistical analysis. A paired two-sided t-test was conducted on protein ratios using a p-value threshold of 0.01. Subsequently, in order to distinguish RIPC specific regulations from random biological variability, an asymmetric normal distribution was drawn from the background ratios (calibrated on the median and ±34.1 percentiles) and only those proteins having a probability <1% to be derived from the background were considered confidently regulated. Finally, protein abundance indexes were estimated using spectral counting where the spectral counting index is simply the number of validated PSMs divided by the protein molecular weight [42].

## Results

**Figure 2:**
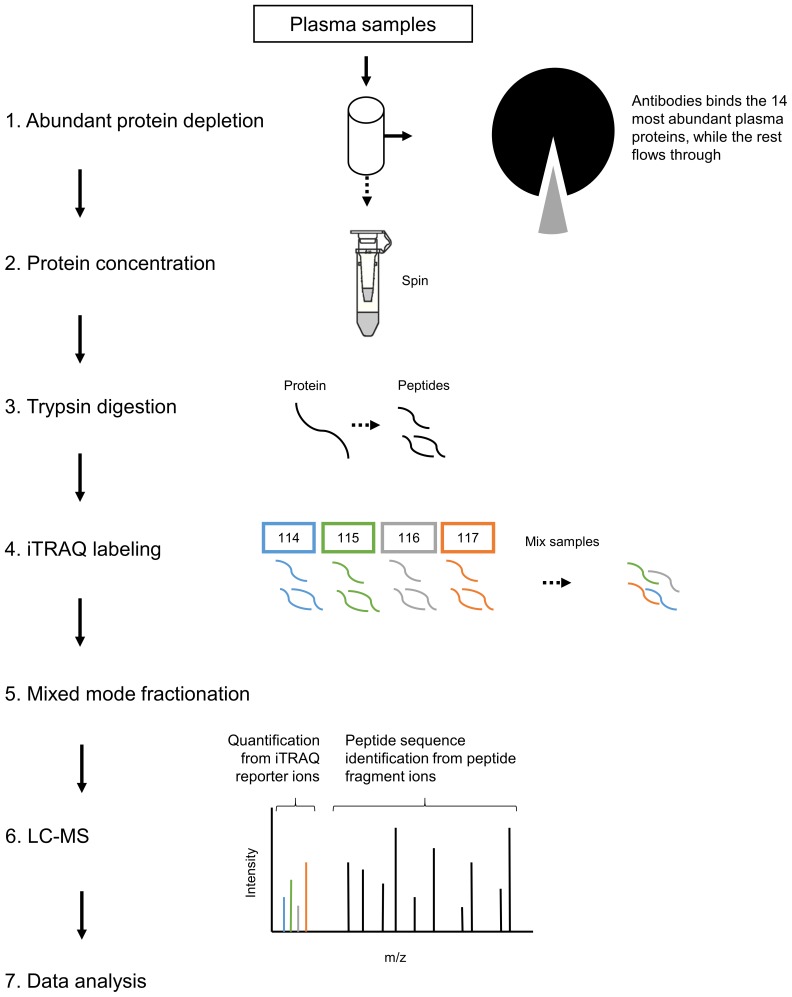
Analysis workflow. Plasma samples were depleted by a MARS Hu-14 column and subsequently concentrated by 3 kDa ultracentrifugation filters. Next, samples were reduced, cysteine blocked and trypsin digested before iTRAQ labeling. The iTRAQ labeled peptides were fractioned into 60 fractions using a mixed-mode reverse phase anion exchanger. Finally, fractions were analyzed on an LTQ-Orbitrap Velos Pro connected to a Dionex Ultimate NCR-3000RS LC system.

**Figure 3:**
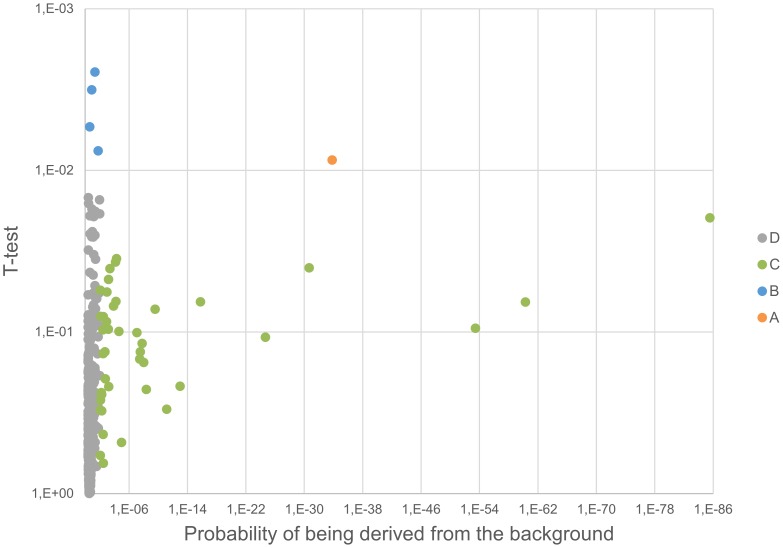
Volcano plot. The significance of the relative regulation of 394 proteins was inspected using (1) a paired two-sided t-test (y axis) and (2) by estimating the probability for the regulation derived from the background (x axis). Proteins are clustered into four categories based on the statistical test passing the threshold (see text for details).

**Figure 4:**
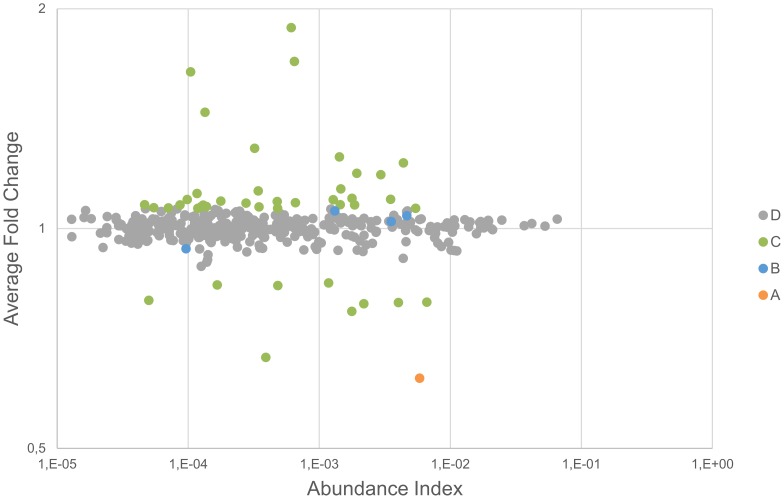
Protein regulation vs. abundance index. The protein regulation is plotted against the abundance index and every protein is classified according to the result of the statistical tests.

The plasma proteome from six healthy donors was compared at baseline and after RIPC as illustrated in Figure 1 and 2. In total, 727 proteins were confidently identified (<1% FDR). Among them, 409 proteins could be accurately quantified by two or more unique peptides. Of these, 393 remained after exclusion of known contaminant and depletion-affected proteins, and are listed in Table S1. These 393 accurately quantified plasma proteins showed accurate and reproducible stability: 388 (98.7%) presented a fold change between 0.91∶1 and 1.1∶1, which is in accordance with the known technical variability of iTRAQ quantification reported in the literature [43]. In order to assess the relevance of the regulations, the quantified proteins were classified into four categories (as detailed in Table S1): (A) proteins with low variability among donors (t-test passed) *and* confident regulation to the background (no protein); (B) low donor variability *but no* confident regulation to the background (4 proteins); (C) high donor variability (t-test failed) but confident regulation to the background (42 proteins); and (D) high donor variability (t-test failed) and no confident regulation to the background (347 proteins). These proteins are plotted in Figure 3 and 4. Notably, no protein was validated by both statistical tests (category A).

Four proteins had a significant p-value (<0.01) between the baseline and RIPC samples for the six subjects examined (category B; Kallistatin, Complement C2, Lactoylglutathione lyase (GLO1), and Cysteine-rich secretory protein 3 (CRISP-3))(Table S1). The fold changes for these, however, were very low (<10% change). We considered changes below 20% to be within what can be explained by the technical variability of the analytical approach. This was corroborated by the statistical analysis, showing no confident regulation to the background. Interestingly, all highly regulated proteins were detected in a much lower abundance (see Figure 4) and failed the t-test (p-value>0.01), suggesting that these cannot be trusted for clinical application.

In contrast, high ratios were systematically found for contaminants and proteins affected by depletion. Remarkably, the remaining amount of proteins targeted by the MARS hu-14 depletion column was consistently less abundant in RIPC samples, hinting at a systematic artifact in the depletion procedure. Among them, fibronectin, previously reported to be regulated after RIPC has been identified in the bound fraction after MARS hu-14 depletion [44]. Fibronectin was confidently regulated to the background (ratios of 0.62∶1) in close agreement with Apolipoprotein A-I (ratio of 0.61∶1) and Haptoglobin (ratio of 0.63∶1), both also targeted by the MARS column. In conclusion, we cannot rule out that the difference in relative abundance measured for these proteins are not simply protocol related. On the other hand, our ability to quantify these potential experimental artifacts demonstrates the reliability of the quantification procedure and rules out the hypothesis that regulations were systematically lost along the workflow.

## Discussion

RIPC of the upper arm is an easy, practical and non-invasive way of inducing protection from ischemia reperfusion induced injury (IRI) in the heart, and the remote ischemic conditioning stimulus can even be applied during or immediately after an ischemic insult (as reviewed in [45]–[47]). The protocol used in the present study was chosen for its proven clinical efficiency: It was demonstrated to reduce the release of troponins (marker for cardiac damage) [3]–[5] and improve survival [6] after coronary artery bypass graft surgery. Shimizu *et al.* used a human RIPC protocol of 4×5 min, sampled venous blood before and after the RIPC intervention, and demonstrated that both whole plasma and plasma dialysate <15 kDa could reduce IRI in the isolated rabbit heart [15]. This protocol is comparable, but not identical, to the one used for the proteomic studies mentioned above, ensuring comparability between the results.

Table S2 gives a generated list of all biomarkers found in the literature, in addition to the ones identified in this study, including the measured ratios and t-test results. As detailed in the introduction, Hepponstall *et al.* [17] report 53 regulated proteins after RIPC. The provided accession numbers were mapped to UniProt entries using the Picr service of the European Bioinformatics Institute [48], leading to mapping of only 14 Swiss-Prot sequences – all other sequences having low or no evidence. 11 of them were confidently quantified in our study (see Table S2). Only Fibronectin (t-test passed) and Haptoglobin (t-test failed) were found to be regulated. However, the regulation of these factors may be experimental artifacts as discussed earlier.

From the 14 proteins reported as regulated by Pang *et al.* [18], 13 could be mapped to Swiss-Prot accessions, and 12 were accurately quantified in our experiment. Among these, only Apolipoprotein A-I was found to be regulated (0.61∶1 ratio). However, as detailed already, it is one of the targets of the depletion procedure. As a result, it is impossible to validate whether this controversial protein is actually regulated by RIPC or whether the up- and down-regulations reported in the literature may be due to experimental artifacts.

Furthermore, we did not quantify SDF-1α, a purported signaling molecule of RIPC [21],[22]. Nor did we identify a single of its peptides. This protein is a good example of the challenges posed when identifying RIPC mediators, as peptide based protein quantification heavily relies on the ability to detect at least two peptides, which can become defying for small proteins. It is important to note that most of the reported potential mediators of RIPC mediated cardioprotection do not meet the mass expectation (<30 kDa). More experimental data are, thus, necessary to assess the role of SDF-1α in RIPC.

In our study, we identified 4 proteins (category B) to be significantly regulated according to the t-test, but showed no confident regulation to the background; Kallistatin, Complement C2, Lactoylglutathione lyase (GLO1), and Cysteine-rich secretory protein 3 (CRISP-3) (Table S1). Despite claiming an overall negative study outcome, as no factors were released in abundance and confidently regulated to the background, it is perhaps timely to consider whether RIPC might be mediated by a consortium of slightly regulated humoral factors, acting on one or several signalling networks, which in concert induce protection. Interestingly, we identify 3 proteins (kallistatin, kallikrein, and kininogen-1) that might inter-relate in the kallikrein-kinin signalling pathway. Of further interest, and adding to the complexity, Complement 2 as part of the complement system may also exerts cross-talk with proteins of the kinin-generating systems. The 4 category B proteins will be discussed further below.

Kallistatin, in particular, proves to be a very interesting candidate in terms of cytoprotective capabilities (UniProt accession P29622, 48 kDa, p-value 0.002, fold-change 1.04). Chao and co-workers identified kallistatin as a tissue kallikrein binding protein (KBP) and a unique serine proteinase inhibitor (serpin) [49]. Later, kallistatin has been ascribed many other functions unrelated to its interaction with tissue kallikrein, including lowering blood pressure, vasodilatation, preventing cardiac remodelling and offering protection against cardiovascular injuries by preventing apoptosis, oxidative stress, and inflammation [50]–[54]. Moreover, the effects of kallistatin seems to be mediated via pro-survival PI3K/Akt/NO dependent signalling, and is postulated to be activated by a yet unidentified kallistatin specific cell surface receptor or binding protein [52]. Kallistatin may also act as an inhibitor of kallikrein [49], and we found kallikrein to be slightly down-regulated in our data (UniProt accession P03952, category D, 71 kDa, p-value 0.014, fold change 0.96). Kallikrein may be activated by lowered plasma pH [55] due to flow restriction imposed by the ischemic conditioning cycles, which in turn can reduce kinin breakdown, enhance bradykinin (BK) formation [56]–[58], and inhibit BK degradation [59]. We also observed that the MK-RPPGFSPFR-SS peptide located at amino-acids 381 to 389 of Kininogen-1 (UniProt accession P01042, catgory D, 72 kDa) was recorded with a 10 times increase in the number of spectra when compared to the median of the peptides for this protein. The peptide may be a product of Kininogen-1 degradation. Plasma and tissue kallikreins converts kiniogens to produce vasoactive kinin peptides, such as bradykinin and lys-bradykinin. BK is known to exert anti-ischemic effects and for being a possible mediator of ischemic preconditioning, although the peptide presence could not directly be related to RIPC in our experiment (ratio of 1.02∶1, p-value of 0.43). But kinin receptors were previously shown to be influenced by RIPC [60], so monitoring kininogen degradation products might be a promising approach elucidating the RIPC mechanism.

The complement system, which is part of the innate humoral immune system, was slightly up-regulated (UniProt accession P06681, 83 kDa, p-value 0.003, fold-change 1.02). Weissman *et al.* [61] demonstrated that complement components are deposited in ischemia reperfused myocardium. In addition, animal models of IR in other organ systems like the gut, kidney, and skeletal muscle indicate that the complement system is a key mediator of IRI [62]–[67]. However, the precise mechanism of complement activation in ischemic tissue has not been clearly elucidated due to the lack of appropriate experimental models, restricted knowledge of the molecular processes causing complement activation during hypoxia in cells, and how it exerts cross-talk between different complement activation pathways [68]. Despite the fact that complement activation during IR is associated with cellular injuries; it is intriguing that the complement system exerts cross-talk with proteins of the kinin generating systems [69].

Lactoylglutathione lyase (also known as glyoxalase I or GLO1) was slightly down-regulated after RIPC (UniProt accession Q04760, 23 kDa, p-value 0.005, fold-change 0.92). Glyoxalase 1 (GLO1) in combination with glyoxalase 2 and the co-factor gluthatione constitute the glyoxalase system, which is responsible for the detoxification of methylglyoxal (MG) [70]. MGs are highly reactive metabolites of glucose degradation pathway, protein and fatty acid metabolism. MG itself is cytotoxic and pro-apoptotic. GLO1 might be a key factor for detoxifying MG and protecting organs against IR injury [71], and may also prevent hyperglycemia-induced diabetic complications [72],[73]. Despite a potential protective role of GLO1, it appears down regulated in our study, and could, thus, be unrelated to the cardioprotective properties of RIPC mediating humoral factors.

Our data also identified glycoprotein human cysteine-rich secretory protein 3 (CRISP-3) as slightly up-regulated (UniProt accession P54108, 28 kDa, p-value 0.007, fold-change 1.06). CRISP-3 is believed to play a role in innate immunity. High levels of human CRISP-3 was found in plasma bound to α_1_-1B-glycoprotein (A1BG-like) (a plasma protein of unknown function) [74]. The A1BG–CRISP-3 complex is thought to inhibit the toxic effect of snake venom metalloproteinases or myotoxins. Udby *et al.* suggests that the A1BG–CRISP-3 complex displays a similar function in protecting the circulation from a potentially harmful effect of free CRISP-3, although the overall function of CRIPS-3 is unclear [74]. Cardiac related effects of CRISP-3 has not been described in the literature as of yet. We also identified the proposed A1BG binding partner in our data (P04217; category D; 54 kDa; p-value 0.16; fold change 1.02).

It is crucial to consider that even with the best analytical approaches available today, there is a limit to how many proteins that can be identified and quantified in plasma. Notably, isoforms, posttranslational modifications and degraded proteins are a vast field of investigation and future experiments might, thus, be directed at other targets in blood. Moreover, we decided to pool all the reperfusion samples for each individual, making sure all potential relevant proteins were present in the sample. However, pooling of the samples may have masked possible time-dependent RIPC induced protein/mediator alterations, in addition to averaging the relative abundance of potential candidate proteins. It also reduces the ability to monitor changes occurring after a single conditioning cycle only. Future work might, thus, improve the time resolution of the experiment.

In addition to technical analytical factors as described here, other aspects of the RIPC procedure such as the number of cycles and stimulus site should be considered when mining for blood borne humoral factors. Loukogeogakis *et al.* elegantly demonstrated a dose-response effect with regards to both site and number of cycles, when exploring for the protective effect of RIPC on endothelial IRI of the arm. The maximum protective effect was obtained with 3 cycles of IR of the arm and at least 2 cycles of the leg [75]. This study is complemented by the study of Hong *et al.*, suggesting that the protective effects of RIPC by lower limb ischemia are greater than those induced by upper limb ischemia [76]. The reason that fewer cycles of the lower limb induced protection may possibly be due to a larger lower limb mass leading to greater release of humoral factors. However, according to a very recent systematic review and meta-analysis [77], it is clear that the optimal RIPC stimulus has not been demonstrated, and it is unknown whether upper or lower limb ischemia is superior. Furthermore, the most favorable timing/duration of the stimulus is unclear. Once the optimal stimulus algorithm, site and timing for the RIPC procedure and more certain indications of the RIPC mediated humoral factor(s) mediating protection are established, it will be timely to compare the healthy plasma RIPC proteome to that of diseased patient populations. Furthermore, comparison of age-matched young and old, male and females might also delineate possible and important age and sex differences.

In conclusion, using shotgun quantification techniques, the detectable portion of the plasma proteomes of six healthy adult males was stable after remote ischemic preconditioning, and in contrast to the literature, no significant changes were identified, other than those that appeared to be related to contaminants or the analytical process itself. Four proteins did, however, show low donor variability (passed the t-test) *but was not* confidently regulated to the background. One way of moving forward could be to increase the workflow sensitivity, with regards to time or biological targets, *e.g.* by selecting on hydrophobicity and size.

## Supporting Information

Table S1 **The complete table of experimental data from all three iTRAQ experiments.** All proteins we identified were classified into four categories based on two statistical tests: (A) proteins with low variability among donors (t-test passed) *and* confident regulation to the background (no protein); (B) low donor variability *but no* confident regulation to the background (4 proteins); (C) high donor variability (t-test failed) but confident regulation to the background (42 proteins); and (D) high donor variability (t-test failed) and no confident regulation to the background (347 proteins).
(XLSX)

Table S2 **A list of all biomarkers found in the literature, in addition to the ones identified in this study.** This includes their measured ratios and t-test results.
(XLSX)
